# Recruiting Diverse Smokers: Enrollment Yields and Cost

**DOI:** 10.3390/ijerph13121251

**Published:** 2016-12-16

**Authors:** Kaitlyn E. Brodar, Marissa G. Hall, Eboneé N. Butler, Humberto Parada, Al Stein-Seroussi, Sean Hanley, Noel T. Brewer

**Affiliations:** 1 Department of Health Behavior, Gillings School of Global Public Health, University of North Carolina at Chapel Hill, Rosenau Hall CB7440, Chapel Hill, NC 27599, USA; kbrodar@email.unc.edu (K.E.B.); mghall@unc.edu (M.G.H.); 2 Lineberger Comprehensive Center, University of North Carolina, Chapel Hill, NC 27599, USA; hparada@live.unc.edu; 3 Department of Epidemiology, Gillings School of Global Public Health, University of North Carolina, Chapel Hill, NC 27599, USA; ebonee@live.unc.edu; 4 Pacific Institute for Research and Evaluation, Chapel Hill Center, Chapel Hill, NC 27514, USA; stein@PIRE.org (A.S.-S.); shanley@PIRE.org (S.H.)

**Keywords:** tobacco, recruitment, low-income, GLB, transgender, African American, Hispanic

## Abstract

To help tobacco control research better include vulnerable populations, we sought to identify effective ways to recruit diverse smokers. In 2014–2015, we recruited 2149 adult cigarette smokers in California and North Carolina, United States, to participate in a randomized trial of pictorial cigarette pack warnings. The most effective means of recruiting smokers were the classified advertising website Craigslist (28% of participants), word of mouth (23%), Facebook (16%), and flyers or postcards (14%). Low-income and African American smokers were more likely to respond to interpersonal contact (including staff in-person recruitment and word of mouth) than were high-income and non-African American smokers (all *p* < 0.05). Hispanic and gay, lesbian, and bisexual smokers were more likely to be recruited by Craigslist than non-Hispanic and straight smokers (both *p* < 0.05). Of the recruitment methods requiring cost, the cheapest was Craigslist ($3–7 per smoker). The most expensive methods were newspaper ads in California ($375 per smoker) and staff in-person recruiting in North Carolina ($180 per smoker). Successfully recruiting diverse smokers requires using multiple methods including interpersonal, online, and other media. Craigslist and word of mouth are especially useful and low-cost ways to recruit diverse smokers.

## 1. Introduction

Despite recent declines in smoking, 17% of adults in the United States (US) smoke cigarettes, a behavior that causes over 480,000 deaths per year [1,2]. Notable disparities in smoking rates exist in the US. Smoking is more common among the poor; gays, lesbians, and bisexuals (GLB); transgender people; and African Americans [2,3,4]. These groups are also disproportionately burdened by smoking-related diseases [5,6,7]. Despite the high rates of smoking in these demographic groups, they are consistently underrepresented in clinical trials [8,9,10,11]. Recruiting smokers from underrepresented and vulnerable populations into tobacco control studies is important for understanding how interventions and policies can reduce smoking disparities.

Multiple studies have examined how to recruit smokers into cessation trials [12]. However, few studies have focused on how to recruit diverse smokers into prevention studies [13,14]. To our knowledge, no studies have explicitly analyzed recruitment of GLB, transgender, or Hispanic smokers, and few have studied how to recruit African American and low-income smokers [13,14]. The literature on cost of recruitment strategies in smoking trials is also limited. Previous studies have found that print materials [15], snowball sampling [16,17], the classified advertising website Craigslist [18], and e-mail invitations [16] may have a low cost per recruited smoker. A direct comparison of the cost per smoker for a wide variety of recruitment strategies, including online, interpersonal methods, and print materials, would be useful to researchers hoping to recruit diverse smokers in a low-cost manner.

We aimed to describe which recruitment strategies were most effective in recruiting diverse smokers for a four-week randomized trial. Additionally, we sought to determine which strategies were more effective in recruiting smokers from groups with disproportionate smoking rates and those underrepresented in research studies. Finally, we aimed to describe the cost per recruited smoker of the recruitment strategies.

## 2. Materials and Methods

### 2.1. Participants

We recruited adult cigarette smokers for a randomized trial of pictorial cigarette pack warnings [19,20] from September 2014 to August 2015 in Chapel Hill, North Carolina (NC) and Oakland, California (CA). Details regarding recruitment, design, and methods of the trial appear in Brewer et al. (2016) [21,22]. The institutional review board at the University of North Carolina approved the study (study number 13-2861). Eligible smokers were ages 18 or older, proficient in English, and current smokers, defined as having smoked at least 100 cigarettes during their lifetime and now smoking every day or some days. Exclusion criteria included pregnancy, concurrent enrollment in a smoking cessation trial, smoking only roll-your-own cigarettes, smoking fewer than seven cigarettes per week, and living in the same household as another trial participant.

### 2.2. Procedures

We used interpersonal, online, and other media recruitment to identify diverse smokers (Table 1). Materials provided a phone number and website. Most (82.6%) smokers completed an eligibility screener online, and 17.4% completed it by phone. We also conducted onsite screenings with interested smokers during staff in-person recruitment efforts. The last page of the online screener stated, “If you are eligible to participate in this study, research staff will contact you soon.” Staff followed up with eligible smokers by email or phone to schedule a baseline appointment. Supplementary Figure S1 provides an example of an advertisement that contains typical wording used in recruitment materials.

### 2.3. Measures

The screening survey assessed how smokers learned about the trial. Response options were the methods listed in Table 1 and an open-ended “other” option. Two coders independently reviewed and coded the open-ended responses yielding two new categories: “other online,” which included responses that indicated the smoker had learned about the trial from websites and applications other than those we used intentionally, and “word of mouth,” which included hearing about the trial from another participant, someone not in the trial, or someone who may or may not have been in the trial. Responses that did not fit into any of the categories remained coded as “other.” The coders met to discuss and resolve disagreements. We used demographic data collected at the baseline appointment after participants enrolled in the trial.

### 2.4. Analysis

We tabulated the number of enrolled smokers recruited by each method, and conducted Pearson chi-square or Fisher’s exact tests to compare low-income to high-income, GLB to straight, transgender to cisgender, African American to non-African American, and Hispanic to non-Hispanic smokers. We estimated total costs for each recruitment method and then calculated the cost per enrolled smoker for each method. Other than staff in-person recruitment costs in North Carolina, which included staff time, we only present direct costs of recruitment materials rather than staff time to develop, manage, and implement the recruitment methods. We did not track cost data for staff in-person recruitment in California. Analyses used SPSS Statistics Version 23.0 (IBM Corp., Armonk, NY, USA). Statistical tests were two-tailed with a critical alpha of 0.05.

## 3. Results

The trial enrolled 2149 smokers, 1186 in California and 963 in North Carolina. The majority of smokers (67%) were between the ages of 25 and 54 (Table 2). Overall, 1.6% identified as transgender, and 17.5% identified as GLB. A large proportion were African American (47.3%) and lived in poverty (54.1%), defined as being at or below 150% of the US federal poverty level [23]; 8.6% were Hispanic.

Craigslist and word-of-mouth recruitment yielded the greatest number of smokers, accounting for 28.0% and 22.9%, respectively, of enrolled participants (Table 3). These methods also accounted for the largest proportion of screened and eligible participants (Supplementary Table S1). Other effective recruitment methods included Facebook (15.6%) and flyers and postcards (13.9%). Email listservs, other online, newspaper ads, and roadside signs each accounted for fewer than 3% of enrolled smokers.

### 3.1. Recruitment Methods by Demographic Group

Low-income smokers were more likely than high-income smokers to be recruited by interpersonal methods and bus ads and less likely to be recruited by Craigslist and roadside signs (all *p* < 0.05, Table 3). Similarly, African Americans were more likely than non-African Americans to be recruited by interpersonal methods (in-person recruiting by staff and word of mouth) and less likely to be recruited by Craigslist, email, and roadside signs (all *p* < 0.05). GLB smokers were more likely than straight smokers to be recruited by Craigslist and Facebook and less likely to be recruited by word of mouth or bus ads (all *p* < 0.05). Transgender smokers were more likely to learn about the trial from flyers or postcards than their cisgender counterparts (*p* < 0.05). Finally, Hispanic smokers were more likely to be recruited by Craigslist and roadside signs, and less likely to be recruited by flyers and postcards (all *p* < 0.05).

### 3.2. Cost per Smoker

Some of the recruitment methods were cost-free, including email listserv, other online, and word of mouth (Table 4). Of these, word of mouth accounted for 22.9% of all participants (Table 3). Free online methods collectively accounted for 2.4% of participants. Of the recruitment methods requiring cost, Craigslist was the lowest cost recruitment method at both trial sites ($7 per smoker in CA and $3 per smoker in NC). Facebook cost $42 per smoker in CA and $62 per smoker in NC, and flyers and postcards cost $84 per smoker in CA and $26 per smoker in NC. Roadside signs, which were only used in NC, were also quite low cost ($33 per smoker). Newspaper and bus ads were the costliest recruitment methods in CA at $375 and $146 per smoker, respectively. In-person recruitment costs were not available from the CA site, but this was the costliest method in NC at $180 per smoker.

## 4. Discussion

Recruiting smokers into research studies can be challenging [24]. We found that Craigslist and word of mouth were the most effective methods overall for recruiting a diverse cohort of smokers. The most effective recruitment method for reaching low-income and African American smokers was word of mouth. However, transgender smokers were most likely to hear about the trial from flyers and postcards. Recruiting a diverse sample of smokers appears to require using an equally diverse set of recruitment approaches. Our paper offers data on how to reach these diverse smokers as well as cost estimates for doing so.

We found that similar approaches worked for recruiting African American and low-income smokers. Word of mouth was the most effective recruitment approach for these groups overall. Another approach that was more effective for these groups than for their counterparts was staff in-person recruiting. Craigslist and roadside signs were less effective recruitment methods for these groups. We are aware of only two other studies that examined methods for recruitment of low-income smokers [13,14] and African American smokers [13]. In one study, Webb and colleagues also found that direct contact was a more effective method for recruiting low-income African American smokers [13]. While we did not examine the effects of mailed invitations or direct phone calls, a recent study found that these two methods were particularly effective at reaching socioeconomically disadvantaged smokers [14]. Other populations in our trial showed substantial variability in what was most effective and more effective in recruitment.

Free recruitment approaches accounted for a large number of enrolled smokers, primarily through word of mouth. Smoking is a social behavior, and smokers are likely to know other smokers [25,26], which may explain the effectiveness of informal word-of-mouth communication as a recruitment method in our trial. Previous studies have paid smokers to refer friends and colleagues. In a study of adolescent smokers recruited at schools and through Facebook, participants who referred an eligible friend that enrolled received a $7 movie ticket [17]. Even though we did not offer an incentive for participant referrals and we did not encourage participants to do so, word of mouth was one of our most effective recruitment strategies, particularly for enrolling low-income and African American smokers. The trial participation incentive and the relative novelty of having pictorial warnings on their cigarette packs, which sparked many social interactions, may have been reasons participants told their friends about the trial [21,25].

Among paid recruitment approaches, Craigslist was uniquely effective at reaching smokers and was also by far the cheapest approach. Facebook was another lower cost approach with respect to cost per recruited smoker. Our findings about cost are consistent with past research showing that web-based advertisements are lower cost than other methods [15,17,18]. For instance, Ramo and colleagues found that Craigslist was particularly low cost per eligible smoker (<$1), although the researchers did not report cost per enrolled participant [18]. Past studies have found that Craigslist is lower cost than Google Adwords, which may cost between $41 and $51 per enrolled smoker [15,16]. Newspaper ads, bus ads, and staff in-person recruiting were all relatively expensive and less effective at recruiting smokers. Rait and colleagues reported a similar pattern to our findings, in which bus ads and school talks (i.e., in-person recruiting) were higher cost than interpersonal referrals and flyers [17]. A major difference was their finding that Facebook ($150/enrolled smoker) was substantially higher cost than flyers ($10/enrolled smoker), whereas we found the costs to be similar ($52 and $55 on average across our trial sites).

Because some costs differed at the two trial sites, cost per recruited smoker also differed between sites for some methods. Flyers and newspaper ads had relatively low cost per smoker enrolled in North Carolina, but these approaches were higher cost in California. Craigslist ads for San Francisco cost $75 per ad, whereas in Chapel Hill, they only cost $25 per ad. However, different yields meant that the Facebook cost per smoker enrolled also differed by site, even though the cost of the advertisements were equal across trial sites. Cost per smoker in the two areas may be driven by differences in how smokers in the two areas access information.

A primary strength of our trial is the diverse sample of smokers recruited using multiple approaches. Few studies have analyzed the utility and cost of a wide variety of strategies for recruiting smokers, and even fewer have focused on recruiting diverse smokers. Another strength was our large sample size, which allowed us to look at comparisons among demographic groups and recruitment methods. Limitations of the trial include missing staff in-person recruiting cost data from the California site, although data were available from the North Carolina site. Another limitation was the relatively small sample of transgender smokers, which limited our ability to detect differences based on gender identity; however, our trial is the first we are aware of that has examined methods for recruitment of transgender smokers. Additionally, we were not able to compare recruitment methods across Hispanic subgroups, as we did not collect data on country of origin. We believe our results may be most applicable to smokers of Mexican origin because they comprise the majority of Hispanics in the areas where we conducted the trial [27]. Similarly, we were not able to look separately at data for American Indians (another group with high smoking rates [28]) because our study included too few to draw meaningful conclusions. Finally, the generalizability of our findings to other geographic areas within the United States remains to be established.

## 5. Conclusions

Researchers interested in recruiting diverse smokers to clinical trials should consider using several recruitment strategies, including Craigslist, word of mouth, Facebook, flyers, and postcards. In particular, Craigslist was effective at recruiting a large number of smokers and was low cost compared to other strategies. Informal word-of-mouth referrals were also an important recruitment strategy, particularly for low-income and African American smokers. In addition, although not low cost, in-person recruitment was effective for reaching low-income and African American smokers. Unlike past studies, we found that word of mouth was effective even when not specifically employed as a snowball sampling strategy that involved payment for referrals. Effectively recruiting diverse smokers for research studies is crucial for understanding the impact of interventions and policies on smoking-related disparities.

## Figures and Tables

**Table 1:** Recruitment Methods.

Method	Description
Interpersonal	
Staff in-person recruiting	In NC, recruiters spent 3–4 h per week recruiting in person at ~80 locations such as bus stops and bars for seven months. In CA, recruiters spent 7–8 h per week recruiting in person at various locations for seven months.
Word of mouth	Did not encourage participants to tell others about the trial. However, some participants told their social contacts about the trial, and other smokers heard about the trial from people who did not participate.
Online	
Craigslist	Placed paid advertisements twice per week on Craigslist website under the “jobs, etc.” section for the San Francisco Bay Area and Raleigh-Durham-Chapel Hill for the duration of the trial.
Facebook	Placed paid advertisements including boosted posts in Facebook newsfeeds for the duration of the trial. The advertisements and posts contained text and pictures of people smoking. Ads targeted adults living near the trial sites and whose profiles included tobacco-related interests.
Email listserv	Sent emails over departmental listservs three times during the trial at local universities in NC.
Other online	Did not recruit through other websites, but some participants noted viewing the study advertisement via other sites and apps.
Other media	
Flyers/postcards	Distributed flyers and postcards to local businesses and organizations including public libraries, restaurants, coffee shops, and bars once per month for seven months. Also provided retailers with postcards to give to patrons at point of sale.
Newspaper advertisements	Ran color display advertisements two days per month and line advertisements six days per month in local newspapers in NC for seven months. Ran display advertisements in local newspapers’ print editions once per week for four weeks and in the online editions every day for one month in CA.
Bus ads	Ran paid advertisements on both the interior and exterior of buses in NC for two months. Ran paid posters at bus shelters in CA for one month.
Roadside signs	Placed “realtor”-style plastic signs on intersections on streets with high traffic volume in NC. Signs were placed at the beginning of the study and were not intentionally removed.

**Table 2:** Participant Characteristics (*n* = 2149).

Characteristic	*n*	(%)
Age		
18–24 years	323	(15.3)
25–39 years	775	(36.7)
40–54 years	642	(30.4)
55+ years	371	(17.6)
Gender		
Male	1039	(48.7)
Female	1060	(49.7)
Transgender	34	(1.6)
Gay, lesbian, or bisexual	368	(17.5)
Hispanic	181	(8.6)
Race		
White	751	(35.7)
Black or African American	994	(47.3)
Asian	70	(3.3)
Other/multiracial	286	(13.7)
Low income (≤150% of Federal Poverty Level)		
No	983	(45.9)
Yes	1159	(54.1)
Trial site		
California	1186	(55.2)
North Carolina	963	(44.8)

Note: Participant characteristics and outcomes at baseline did not differ by trial arm. Missing demographic data ranged from 0.7% to 2.2%.

**Table 3:** Yield of Priority Demographic Groups for Recruitment Methods.

Recruitment Method	Overall	Income		Race		Ethnicity		Sexual Orientation		Gender Identity	
		High-Income	Low-Income	Not African American	African American	Not Hispanic	Hispanic	Straight	GLB ^b^	Cisgender	Transgender
	(*n* = 2149) %	(*n* = 983) %	(*n* = 1159) %	(*n* = 1107) %	(*n* = 994) %	(*n* = 1935) %	(*n* = 181) %	(*n* = 1734) %	(*n* = 368) %	(*n* = 2099) %	(*n* = 34) %
Interpersonal											
In-person recruiting	5.4	3.1	7.5 **	3.9	6.7 *	5.4	5.5	5.8	4.1	5.4 ^a^	2.9
Word of mouth	22.9	14.8	29.7 **	13.5	33.0 **	23.0	18.2	23.4	17.4 *	22.7	20.6
Online											
Craigslist	28.0	39.4	18.4 **	37.7	17.5 **	26.9	42.5 **	27.2	33.4 *	28.3	20.6
Facebook ad	15.6	18.8	13.0 **	16.1	15.1	15.8	15.5	15.1	19.3 *	15.5	26.5
Email listserv	1.7	1.9	1.5	2.6	0.7 **	1.7 ^a^	1.7	1.4	2.7	1.7 ^a^	0.0
Other internet	0.7	0.6	0.8	0.9	0.5	0.7 ^a^	1.1	0.8 ^a^	0.3	0.7 ^a^	0.0
Other media											
Flyer/postcard	13.9	12.4	14.7	13.6	14.3	14.7	4.4 **	13.4	14.4	13.5 ^a^	29.4 *
Bus ad	4.4	2.4	6.1 **	3.7	5.2	4.4	2.8	5.0	1.9 *	4.4 ^a^	0.0
Newspaper ad	2.6	2.0	3.1	2.6	2.6	2.7 ^a^	2.2	2.7	2.7	2.7 ^a^	0.0
Roadside sign	1.7	2.5	1.0 *	2.4	0.9 *	1.5 ^a^	3.9 *	1.9	1.1	1.7 ^a^	0.0
Other	0.8	0.5	1.2	0.8	1.0	1.0 ^a^	0.0	0.8 ^a^	1.4	0.9 ^a^	0.0
Missing	2.4	1.5	3.1 *	2.3	2.4	2.4	2.2	2.5	1.4	2.4	0.0

* *p* < 0.05, ** *p* < 0.001; ^a^ Fisher’s exact test used due to small cell sizes; ^b^ GLB = Gay, lesbian, or bisexual. Note: Missing demographic data ranged from 0.7% to 2.2%.

**Table 4:** Recruitment Costs.

Recruitment Method	California		North Carolina	
	Total Cost	Cost per Enrolled Smoker	Total Cost	Cost per Enrolled Smoker
Interpersonal				
In-person recruiting ^a^	-	-	$10,074	$180
Word-of-mouth	$0	$0	$0	$0
Online				
Craigslist	$3500	$7	$350	$3
Facebook ad	$9000	$42	$7557	$62
Email listserv ^c^	-	-	$0	$0
Other online	$0	$0	$0	$0
Other media				
Flyer/postcard	$8500	$84	$5128	$26
Newspaper ad	$6000	$375	$2997	$75
Bus ad ^b^	$3500	$146	$6188	$87
Roadside sign ^c^	-	-	$1191	$33
Other	-	-	-	-

^a^ Data missing from CA; ^b^ In NC, these were interior and exterior signs on buses. In CA, these were ads inside of bus stop shelters; ^c^ Email listserv and roadside signs were not used in CA.

## Supplementary Materials

The following are available online at www.mdpi.com/1660-4601/13/12/1251/s1, Figure S1: Recruitment advertisement includes an image of the general advertisement wording used in recruitment materials. Table S1: Recruitment Method for Screened, Eligible, and Enrolled Participants includes the percentage of screened, eligible, and enrolled smokers that we identified by each recruitment method.
