# Supplementary material for: Recruiting Diverse Smokers: Enrollment Yields and Cost
Source: Int J Environ Res Public Health. 2016 Dec 16;13(12):1251. doi: 10.3390/ijerph13121251 (PMC5201392; doi:10.3390/ijerph13121251)
Supplement: Supplementary file 1 [file ijerph-13-01251-s001.pdf]

Supplementary Materials: Recruiting Diverse Smokers: Enrollment Yields and Cost

Kaitlyn E. Brodar, Marissa G. Hall, Eboneé N. Butler, Humberto Parada, Al Stein-Seroussi, Sean Hanley and Noel T. Brewer

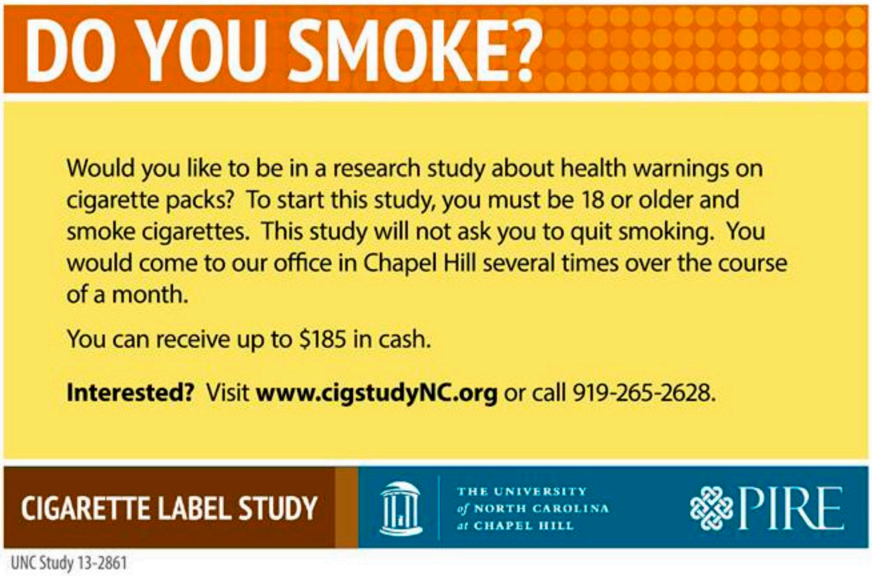

Figure S1. Recruitment advertisement. Note: Exact wording varied slightly depending on recruitment method.

Table S1. Recruitment method for screened, eligible, and enrolled participants.

Recruitment Method	%		
	Screened	Eligible	Enrolled
n	6732	5731	2149
Interpersonal			
In-person recruiting	4.3	4.2	5.4
Word of mouth	14.3	14.4	22.9
Online			
Craigslist	31.9	32.4	28.0
Facebook ad	26.8	25.8	15.6
Email listserv	2.1	1.9	1.7
Other online	1.3	1.2	0.7
Other Media			
Flyer/postcard	9.4	9.7	13.9
Bus ad	3.5	3.7	4.4
Newspaper ad	2.2	2.4	2.6
Roadside sign	1.7	1.7	1.7
Other	0.7	0.6	0.8
Missing	1.9	2.0	2.4

© 2016 by the authors; licensee MDPI, Basel, Switzerland. This article is an open access article distributed under the terms and conditions of the Creative Commons by Attribution (CC-BY) license (http://creativecommons.org/licenses/by/4.0/).
